# Supplementary material for: Model-Based Dielectric Constant Estimation of Polymeric Nanocomposite
Source: Polymers (Basel). 2022 Mar 11;14(6):1121. doi: 10.3390/polym14061121 (PMC8955738; doi:10.3390/polym14061121)
Supplement: Supplementary file 1 [file polymers-14-01121-s001.zip › polymers-1614436-supplementary.pdf]

Supporting Information

Model-based dielectric constant estimation of polymeric nanocomposite

Jiang Shao[1], Le Zhou [2], Yuqi Chen[3], Xue Liu[4,*] and Mingbo Ji[5,*]

[1]Jiangsu University of Science and Technology, Zhenjiang, 212100, P. R. China; Jiang-Shao@hotmail.com
[2]Changzhou Betterial Film Technologies Co., Ltd., Changzhou, 213000, P. R. China; zhoule@ betterial.cn
[3] University of Pennsylvania, Philadelphia, 19104, US; sevenchen9819@gmail.com
[4] Henan Key Laboratory of Polyoxometalate Chemistry, College of Chemistry and Chemical Engineering, Henan University, Kaifeng, Henan 475004, P. R. China; xliu@ipe.ac.cn
[5] Harbin Engineering University, 264000 Yantai, China; minjikt@outlook.com
* Correspondence: xliu@ipe.ac.cn; Tel.: +86 15101544706; minjikt@outlook.com; Tel.: +86 13041090299

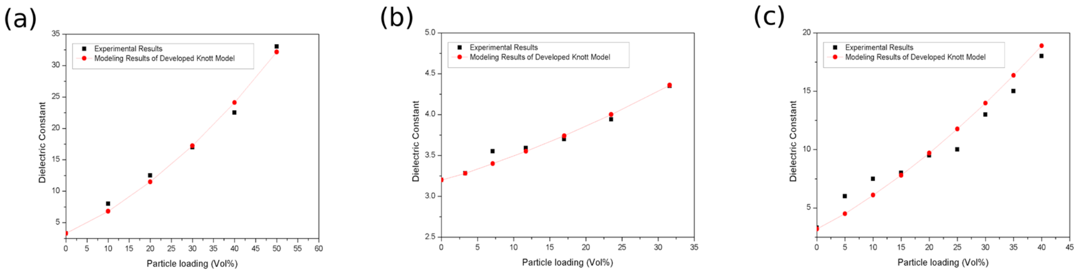

Figure S1 The dielectric constant of the experimental data and modeling results based on the developed Knott model of three nanocomposites: (a) PI-$BTiO_3$ composite; [1] (b) ER-Al_2O_3 composite; [2] (c) PI-$BTiO_3$ composite [3]

Reference

1. Fan B H, Zha J W, Wang D R, et al. Experimental study and theoretical prediction of dielectric permittivity in $BaTiO_3$/polyimide nanocomposite films[J]. Applied Physics Letters, 2012, 100(9): 092903.

2. Kochetov R, Andritsch T, Morshuis P H F, et al. Thermal and electrical behaviour of epoxy-based microcomposites filled with Al_2O_3 and SiO_2 particles[C]//2010 IEEE International Symposium on Electrical Insulation. IEEE, 2010: 1-5.

3. Dang Z M, Lin Y Q, Xu H P, et al. Fabrication and dielectric characterization of advanced $BaTiO_3$/polyimide nanocomposite films with high thermal stability[J]. Advanced Functional Materials, 2008, 18(10): 1509-1517.
